# Assessment of Land-Use and Land-Cover Change in Guangxi, China

**DOI:** 10.1038/s41598-019-38487-w

**Published:** 2019-02-18

**Authors:** Yunfeng Hu, Batunacun, Lin Zhen, Dafang Zhuang

**Affiliations:** 1 State Key Laboratory of Resources and Environmental Information System, Institute of Geographic Sciences and Natural Resources Research, Chinese Academy of Sciences, Beijing, 100101 China; 2 University of Chinese Academy of Sciences, Beijing, 100049 China; 3 Department of Geography, Humboldt–Universität zu Berlin, Unter den Linden 6, 10099 Berlin, Germany; 4 Leibniz Centre for Agricultural Landscape Research (ZALF), Eberswalder Straße 84, 15374 Müncheberg, Germany

## Abstract

It is increasingly acknowledged that land-use and land-cover change has become a key subject that urgently needs to be addressed in the study of global environmental change. In the present study, supported by the long-time-series of land-use and land-cover data from 1990, 2000, and 2017, we used the land-use transition matrix, Markov chain model and Moran’s I to derive detailed information of the spatial patterns and temporal variation of the land-use and land-cover change; additionally, we highlight the deforestation/afforestation conversion process during the period of 1990–2017. The results show that a total of 4708 km^2^ (i.e., 2.0% of the total area) changed in Guangxi from 1990 to 2017, while 418 km^2^ of woodland has been lost in this region. The woodland lost (deforestation) and woodland gained (afforestation) were collocated with intensive forest practices in the past 27 years. The conversions from woodland to cropland and from woodland to grassland were the dominant processes of deforestation and afforestation, respectively. Steep slope cropland was one of the major conversion patterns of afforestation after 2000. This result is mainly explained by the implementation of the “Grain for Green Program” policy and the large-scale development of eucalyptus plantations. Further efforts should be made to control deforestation in this area. These findings can also be used as a reference in the formulation and implementation of sustainable woodland management policies.

## Introduction

Land-use and land-cover change (LUCC) has been considered an important research topic for global environmental change and sustainable development^1–7^. As an important part of worldwide sustainable development, LUCC and deforestation in China has attracted great attention^8^. Land cover refers to the biophysical attributes of the Earth’s surface, while land use refers to the human purpose or intent applied to these attributes^9^. Many previous studies have focused on revealing the effects of LUCC, e.g., the significant effects on resource production and the influence on climate change^10^, soil erosion^11^, biodiversity^4^, food security^4^, and even threats to public health^12^, while also concentrating on determining the drivers of LUCC^13,14^. However, examining the process and trends of LUCC via quantitative analysis is a prerequisite to gain a deeper understanding of LUCC and help policy makers set improvement targets in specific areas and adopt appropriate practices while also keeping in line with other fields of sustainability^1,10,15,16^. However, land-use data are also a prerequisite issue for the evaluation of LUCC in a specific region^17,18^. Based on this, we used land-use and land-cover datasets due to their high accuracy and long time scale features, which were produced by Chinese academics, to identify the patterns and processes of LUCC in the study region^19–21^.

Deforestation is an important process of LUCC^10^, and it is also a factor that provides feedback for the drivers of land-use change^22^. Many previous studies have reported that deforestation has a close relationship with climate change^23,24^, biodiversity loss and increases in CO_2_ and other greenhouse gases^8,25^, soil degradation^26^, flooding^27^, ecosystem services^28^, and human livelihood^29^. The effects of LUCC can even threaten economic development and food security^30,31^. Therefore, understanding the deforestation process and the related drivers in a quantitative way is necessary to provide appropriate policy intervention. The International Panel on Climate Change (IPCC) emphasized that the conversion between forest and other land-use types (e.g., forest to cropland, grassland, or other land-use types) is frequently referred to as deforestation. In contrast, afforestation “occurs when forest cover expands through the planting of trees on lands without trees”^32^. Thus, in the present study, deforestation is defined as woodland being converted into other land-use types, while afforestation is defined as other land-use types being changed into forested areas. The drivers of deforestation have significant regional characteristics, with the most common drivers of deforestation summarized as logging for timber, commercial agricultural development, urban and mining expansion, and grazing in Asia^33^. The implementation of a set of forest protection policies has contributed significantly to the growth of forest cover in China during the last 20 years^34^. Severe deforestation previously occurred in China, and the temporal and spatial characteristics of deforestation^35^, the drivers of deforestation^36^, and the conservation policies^37^ have been reported in several past studies^36–39^. However, there is a distinct lack of studies examining the transition between other land-use types and deforestation/afforestation based on a long-time-series of land-use data.

Since the economic reformation in China, land use has changed significantly, with various patterns and driving forces found at different spatial scales^20,21,40^. An LUCC analysis using long-time-scale land-use data is effective for determining the major land-use conversion and provides useful information for planners and policymakers. Guangxi was chosen as a case study, as the southern area of China has experienced conflict between urbanisation and basic arable land protection as well as conflict between basic arable land protection and woodland protection in the past several decades^3,41,42^. The acceleration of urbanisation and industrialisation has led to serious ecological destruction, such as a decrease in ecological carrying capacity, vast coverage and intensity of water and soil loss, desertification, soil erosion, vegetation degradation, biodiversity losses, invasion of alien species, environmental pollution, natural hazards, geological hazards, and forest hazards^43,44^. Considering these conditions, the objectives of this research are as follows: (1) to identify the land-use change process in Guangxi during the periods of 1990–2000 and 2000–2017; (2) to evaluate the deforestation patterns based on LUCC analysis, and (3) to discuss the relationship between deforestation/afforestation patterns and possible drivers from a policy perspective.

## Results

### Trend of LUCC since 1990

**Figure 1:**
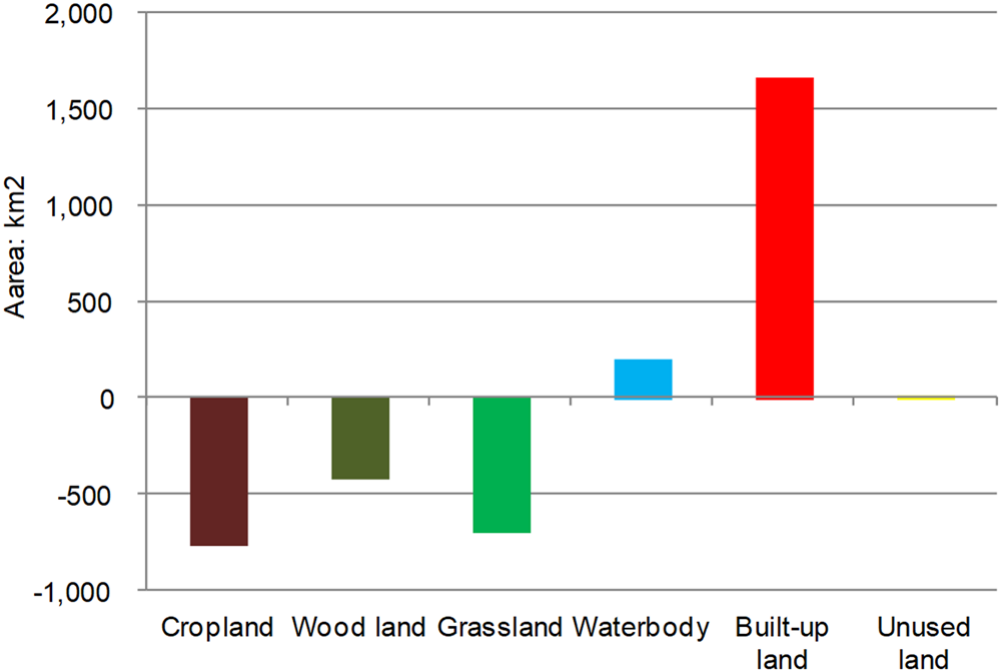
The land-use and land-cover change (LUCC) net change during the period of 1990–2017.

**Figure 2:**
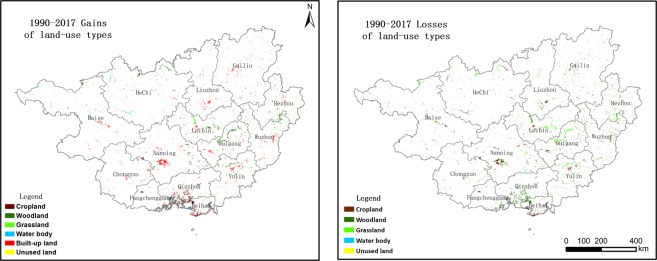
The spatial pattern of land-use change in Guangxi (1990–2017), including gains (left) and losses (right) for specific land-use types.

The land use net change and spatial patterns are presented in Figs 1 and 2. From 1990 to 2017, a total of 4708 km^2^ (2.0% of the total area), including the six investigated land-use types, underwent changes in Guangxi. As seen in Fig. 1, the land-use types that increased the most were built-up land and water bodies, which increased by 1656 km^2^ (0.8% of the total area) and 197 km^2^ (0.08% of total area), respectively, since 1990. Every municipality expanded during the past 27 years (Fig. 2). In contrast, the cropland and grassland areas decreased significantly in this period, shrinking by 753 km^2^ (0.32%) and 686 km^2^ (0.29%), respectively, in the study area; additionally, cropland gains (703 km^2^) occurred in Qinzhou. This decrease was followed by woodland areas, which decreased by 418 km^2^. Unused land was only altered slightly, with a change of less than 0.01% in the total area (8.6 km^2^), and woodland gains (1362 km^2^) occurred mostly in Guigang, Wuzhou, and Yulin. The results suggest that the area of land types with higher ecological values (e.g., woodland, grassland, and cropland) decreased. Water bodies showed a slight increase in this region, and the major driver can be summarised as the national water sources/river protection polices that were implemented in the 1990s. However, because most remote sensing images were obtained from similar seasons (July–October), which includes the flood season in the southern region, this can also lead to interpolation errors for water bodies.

### The Temporal and Spatial Variation Trend of LUCC since 1990

**Figure 3:**
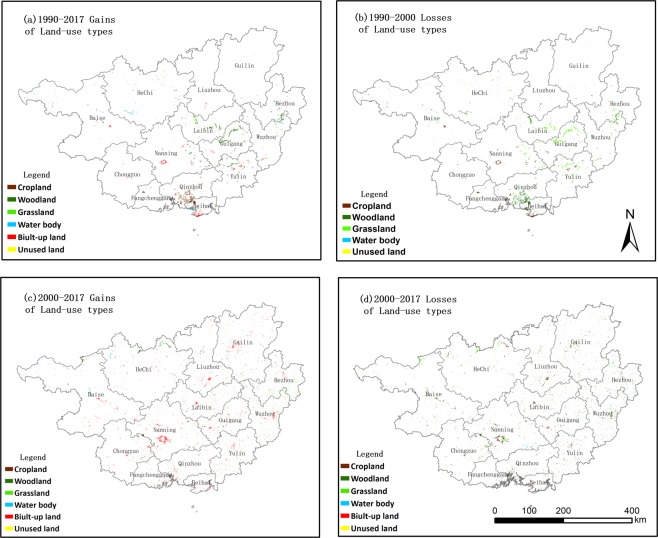
The spatial pattern of land-use change in Guangxi, including gains (left) and losses of specific land-use types. (**a**) 1990–2000 gains, (**b**) 1990–2000 losses, (**c**) 2000–2017 gains, and (**d**) 2000–2017 gains.

**Figure 4:**
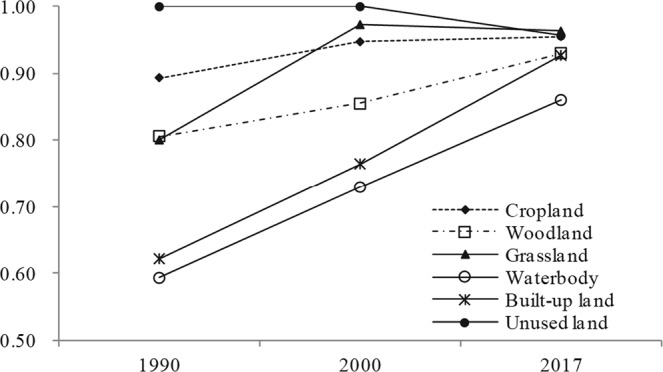
Moran’s I of land use and land cover in Guangxi between 1990 and 2017.

The land-use change processes and patterns are presented with the transition matrix and TPs (see Tables 1–3). The spatial characteristics and spatial autocorrelation of LUCC are presented in Figs 3 and 4.

**Table 1:** Transition matrix of LUCC in Guangxi between 1990 and 2017 (unit: km^2^).

Periods	Land-use types	Cropland	Woodland	Grassland	Water body	Built-up land	Unused land	Losses
1990–2000	Cropland	51,246	70	4	65	356	0	495
	Woodland	496	154,962	157	111	35	0	799
	Grassland	68	673	20,813	7	8	0	757
	Water body	15	7	2	3687	23	0	47
	Built-up land	0	0	0	0	4167	0	0
	Unused land	0	0	0	0	0	36	0
	Gains	580	750	163	184	421	0	2098
2000–2017	Cropland	50,846	193	24	70	692	1	980
	Woodland	112	154,704	422	64	400	8	1008
	Grassland	12	419	20,400	29	117	0	576
	Water body	18	28	37	3,762	25	2	109
	Built-up land	0	0	0	0	4589	0	0
	Unused land	0	0	0	1	1	34	3
	Gains	142	640	484	165	1235	11	2676

**Table 2:** Transition possibilities between 1990 and 2000.

	Cropland	Woodland	Grassland	Water body	Built-up land	Unused land
Cropland	99.04	0.14	0.01	0.13	0.69	0.00
Woodland	0.32	99.49	0.10	0.07	0.02	0.00
Grassland	0.32	3.12	96.49	0.03	0.04	0.00
Water body	0.41	0.17	0.05	98.74	0.62	0.00
Built-up land	0.00	0.00	0.00	0.00	100.00	0.00
Unused land	0.01	0.05	0.00	0.14	0.00	99.80

Note: Built-up land in this region cannot be changed into other land-use types; thus, the TP of built-up land is 100, while unused land is the smallest land-use type, occupying only 0.15% of the entire study area. Therefore, in this study, we did not analyse this land-use type. The same applies to Table 3.

**Table 3:** Transition possibilities between 2000 and 2017.

	Cropland	Woodland	Grassland	Water body	Built-up land	Unused land
Cropland	98.11	0.37	0.05	0.14	1.34	0.00
Woodland	0.07	99.35	0.27	0.04	0.26	0.01
Grassland	0.06	2.00	97.25	0.14	0.56	0.00
Water body	0.47	0.72	0.95	97.17	0.64	0.05
Built-up land	0.00	0.00	0.00	0.00	100.00	0.00
Unused land	0.07	1.15	0.01	3.62	2.59	92.57

During 1990–2000, all land-use type activates remained relatively stable with TPs larger than 0.96. Table 2 indicates that the most stable land-use types were woodland and cropland, followed by water bodies and grassland. The activities of all land-use types were slight in this period; for example, the woodland was the dominant land-use type, but a total of 799 and 750 km^2^ of woodland changed into or was converted from other land-use types.

There were four major land-use changes that progressed as follows: built-up land expansion, deforestation, afforestation and decreased grassland. First, there was an expansion of built-up land (421 km^2^, also called urbanisation and industrialisation) (see Table 1), and a total of 356 km^2^ of built-up land was converted from cropland. The most obvious conversion occurred in the south of Guangxi (e.g., Beihai, Nanning, and Yulin) (Fig. 3a). Second, there was deforestation (woodland changed into other land-use types), and a total of 799 km^2^ of woodland was changed into other land-use types. Furthermore, deforestation was also accompanied by an expansion of cropland, and a total of 580 km^2^ of cropland was gained from other land-use types, with approximately 85.5% (496 km^2^) converted from woodland. Figure 3b shows that the most obvious deforestation occurred in Guigang, Yulin, Laibin, and Wuzhou. Deforestation and afforestation (increased woodland) co-existed during this period. There was an 750 km^2^ increase in the woodland area, which mostly occurred in Guigang, Yulin, and Laibin (Fig. 3a). Afforestation was also accompanied by a decrease in grassland, and approximately 757 km^2^ of grassland was changed into other land-use types; moreover, approximately 88.9% (673 km^2^) was converted into woodland. Figure 3b shows that the most obvious decrease in grassland occurred in Guigang, Yulin, Laibin, and Wuzhou. In this period, 184 km^2^ of water bodies were transformed from other land-use types. At the same time, 47 km^2^ of water bodies were changed into other land-use types. There was a slight decrease in unused land in this area.

During 2000–2017, land-use activities became slightly stronger, with the lowest TPs of 0.97 (see Table 3). Woodland was still the most stable land-use type with TPs of 99.35%. In this period, a total of 1007 and 640 km^2^ of woodland was lost or gained, respectively, in Guangxi.

Three different land-use change processes were identified in Guangxi. Built-up land continued to expand in this period, with an increase of 1235 km^2^ (see Table 1). All 14 cities in Guangxi experienced dramatically accelerated urbanisation and industrialisation processes after 2000 (Fig. 3c). In this period, built-up expansion was accompanied by cropland shrinkage, and 980 km^2^ of cropland was changed into other land-use types, while approximately 70.6% (692 km^2^) was changed into built-up land. The greatest decrease in cropland occurred in Nanning, Baise, Guilin, and Guigang (Fig. 3d). Deforestation and afforestation continued to co-exist in this period. The total area covered by woodland decreased by 1008 km^2^, and most woodland was changed into grassland (422 km^2^) and built-up land (400 km^2^). Deforestation mostly occurred in Wuzhou, Hezhou, Nanning, and Baise. In contrast, woodland increased significantly by 640 km^2^, with approximately 65.5% (419 km^2^) transformed from grassland. Figure 3d shows that most of the afforestation was located in Hechi, Baise, Laibin, and Nanning. In this period, water bodies continued to increase by 56 km^2^, with 109 km^2^ of the water body area having changed into other land-use types and 165 km^2^ having been converted to water bodies from other land-use types (see Table 1).

### Spatial Autocorrelation of LUCC in Guangxi

In a bid to explore the spatial patterns dependence via land use data, the global spatial autocorrelation of each land-use type is presented in Fig. 4. Moran’s I of woodland, built-up land and water bodies increased from 1990 to 2017, of which the spatial autocorrelation of woodland and water bodies was stronger in Guangxi. The spatial autocorrelation of built-up land is also stronger, mainly due to the rapid urbanisation and industrialisation in Guangxi. At the same time, Moran’s I of grassland, cropland and unused land initially increased and then decreased after 2000. In other words, the spatial autocorrelation of these land-use types has weakened since 2000.

### Deforestation and Afforestation in Guangxi since 1990

**Figure 5:**
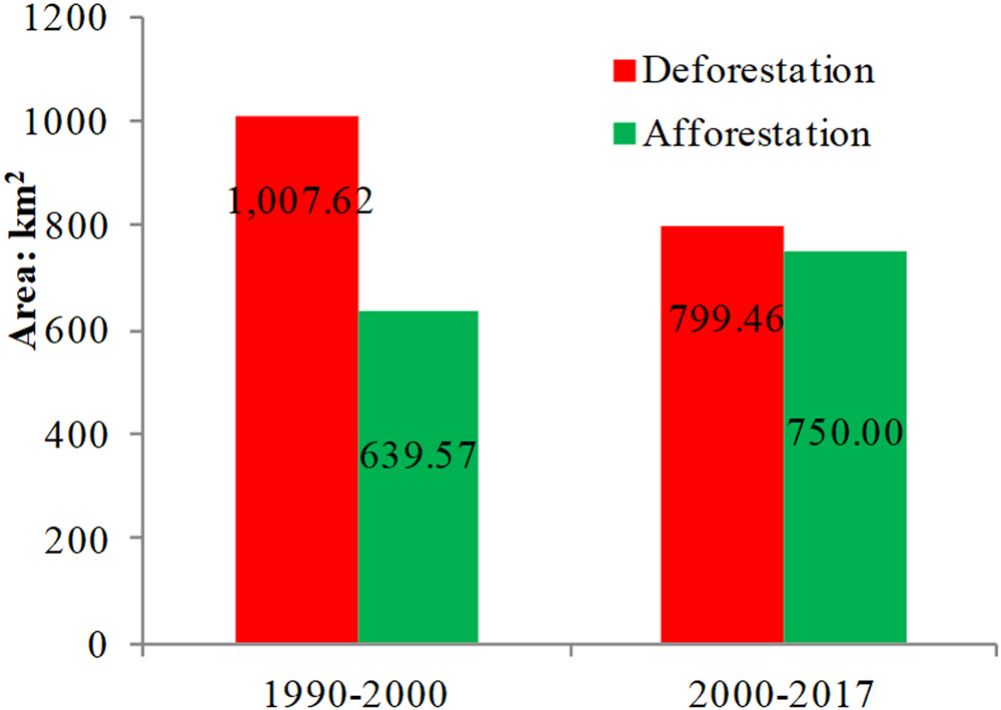
Deforestation and afforestation obtained from Table 2 (1990–2000 and 2000–2017).

**Figure 6:**
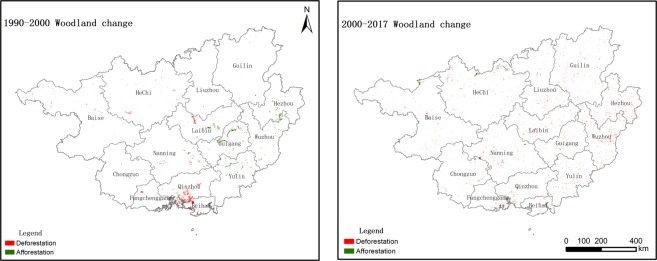
The map of deforestation and afforestation in Guangxi (left: 1990–2000, right: 2000–2017). Note: deforestation refers to woodland changed into other land-use types, e.g., woodland converted into cropland, grassland, etc.; afforestation refers to other land-use types changing from other land-use types. Please see Supplementary Fig. S1.

Woodland is the largest land-use type in Guangxi and experienced a different conversion process during 1990–2017. In the period of 1990–2000, 799 km^2^ of woodland was changed into other land-use types, with the deforestation process mainly including one conversion pattern: woodland converted into cropland (see Table 2, Figs 5 and S1). Figure 6 shows that the most obvious deforestation occurred in the coastal areas, followed by the middle area and the other eastern area cities. During this period, 750 km^2^ of afforestation occurred in Guangxi, with the major afforestation pattern being the conversion of grassland into woodland (Tables 2 and 3). The most obvious afforestation occurred in the middle and eastern areas. During this period, woodland exhibited an overall trend of deforestation.

During 2000–2017, deforestation continued, and the deforestation mainly included two patterns in this period: woodland changed into grassland and woodland converted into built-up land (see Tables 2 and S1). The transformation of woodland to cropland was effectively curbed after 2000. At the same time, the major afforestation patterns were the conversion of cropland and grassland into woodland (see Tables 2 and S1). Figure 6 shows that the most obvious deforestation occurred in Wuzhou, Hezhou, Nannig, and Baise. At the same time, the most obvious afforestation occurred in Hechi, followed by Baise, Laibin, and Nanning. During the past 27 years, deforestation and afforestation have co-existed in Guangxi. However, the area of deforestation decreased, and the area of afforestation increased compared with the earlier period (Fig. 5).

### Spatial-temporal patterns of cropland conversion in Guangxi

The feature of the “Grain for Green Program” (GFGP) (also known as the “Sloping land conversion program”) is the conversion of steep-slope or degraded cropland into forest or grassland^45^. In a bid to evaluate the GFG effects, in this study, we calculated the converted cropland at different slope gradients. The GFG regulations and previous studies have suggested that slopes greater than 15 degrees or 25 degrees were encouraged to be converted to forest^46,47^. In a bid to analyse the conversion cropland response to GFGP policies and patterns of deforestation and afforestation, in this study, we calculated the slope gradient of converted cropland, as well as deforestation and afforestation. Using 90-m STRM DEM data (http://srtm.csi.cgiar.org/SELECTION/inputCoord.asp), the map of slope in the study area was processed by ArcGis software. The slope was classified into four levels by thresholds of 2, 6, 15, and 25 degrees^46^. The converted cropland and the total areas of deforestation and afforestation were identified by different slope degrees.

There was a total of 51,816.9 km^2^ of cropland in Guangxi in 2000 (Table 2). Of this cropland, 72.7% were distributed on slope gradients less than 6 degrees, 16.8% were distributed on slopes between 6 and 15 degrees, 7.6% were distributed between 15 and 25 degrees, and approximately 3% were located on slopes greater than 25 degrees (see Table 4). Regarding cropland that was converted to woodland on different slope gradients, in here we used the area of the cropland that was converted to woodland in each slop gradient, divided by the total area of cropland in each slop gradient. The results indicate that 0.71% of was converted on slopes between 15 and 25 degrees, 0.47% was converted on slopes greater than 25 degrees, and 0.56% was converted on the slopes between 5 and 15 degrees. Overall, comparing cropland that was located in different slop gradients, croplands with slopes greater than 15 degrees were the primary category of converted croplands.

**Table 4:** Cropland conversion on different slope gradients (between 2000 and 2017). (cropland converted to woodland).

Slope gradient	Percent of cropland that converted to woodland in different slope gradients	Percent of cropland in different slope gradients
0–2	0.25%	44.77%
2–6	0.34%	27.94%
6–15	0.56%	16.75%
15–25	0.71%	7.59%
>25	0.47%	2.95%

## Discussion

### The drivers of woodland change from a policy perspective

#### The deforestation/afforestation patterns and relations with possible drivers before 2000

In this research, based on long-time-series and high accuracy land-use data (Chinese National Land use/Land Cover Database, CNLCD), we used the land-use matrix method, Markov chain model and Moran’s I to derive detailed LUCC information and analyse the deforestation and afforestation conversion patterns between two periods (1990–2000 and 2000–2017).

**Figure 7:**
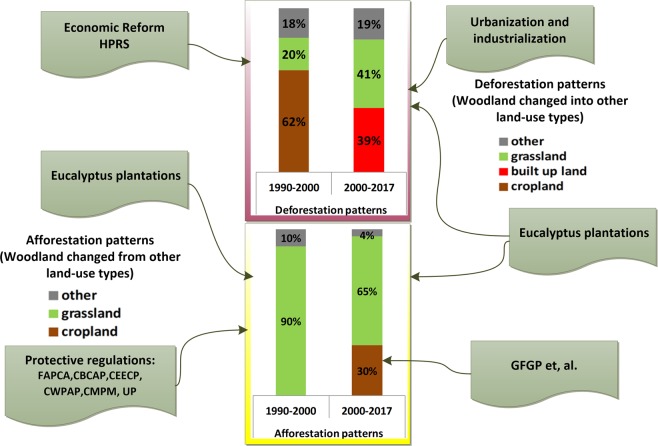
The relationships between possible policies and deforestation/afforestation patterns. Note: deforestation pattern: woodland conversion into other land-use types; afforestation pattern: other land-use type changed into woodland.

During the past 27 years, woodland has experienced several conversion patterns in Guangxi. In the period of 1990–2000, woodland changed into cropland (deforestation), and grassland was converted into woodland (afforestation) (see Fig. 7). The deforestation process was accompanied by cropland expansion, with cropland expansion being a major driver of deforestation worldwide^42,48,49^. In China, approximately 50% of cropland expansion resulted from deforestation in the past 300 years^50^, and this global value is 60%^10^. In China, the Economic Reform began at the end of the 1970s, in which cropland demand increased dramatically to feed the large population and support the agricultural industries^51^. Since 1979, the Household Production Responsibility System (HPRS) has been in effect, and in 1992, the market-directed economic system launched in China. All of these policies have led to specific land-use conversion, such as the conversion of woodland into cropland and built-up land^41,42,52^.

However, afforestation at the expense of grassland is mainly due to several important protective regulations that were launched in this period. During the 1990s, in a bid to protect woodland areas in China, a set of regulations were launched, including the China Biodiversity Conservation Action Plan (1994) (CBCAP)^53^, the Forestry Action Plan for China’s Agenda 21 (1995) (FAPCA)^54^, the China Ecological Environment Conservation Plan (1998) (CEECP), the China Wetland Protection Action Plan (2000) (CWPAP), the China Mangrove Protection Management (CMPM), and the Utilization Plan (2002) (UP)^52^. All of these regulations and policies promoted afforestation throughout this period. During this period, approximately 20% of converted woodland was changed into grassland, and at the same time, approximately 41% of converted woodland was changed from grassland in Guangxi (see Fig. 7). One possible important driver of the conversion pattern is the development of eucalyptus plantations. Since the early 19th century, eucalyptus has been imported from France and is widely distributed throughout Guangxi; additionally, eucalyptus is the largest production base of wood in China, and eucalyptus has shown a significant contribution to local economic development and the timber industry^55^. Eucalyptus forests include features such as high yields, annual forest growth, and annual harvest volume; moreover, this rapid growth and harvest is also evident from remote sensing data as an alternative to grassland and woodland.

#### The deforestation/afforestation patterns and relations with possible drivers after 2000

After 2000, the deforestation pattern involved the conversion of woodland into built-up land (39% of converted woodland, see Fig. 7) and grassland (41% of converted woodland). The rapid development of urban areas in southern China has contributed significantly to deforestation. Urbanisation and industrialisation are necessary stages in the development of social and economic systems. However, the unprecedented combination of economic reconstruction and population growth has led to a change in the country’s societal structure, from a largely rural society into a predominantly urban society. A total of 670 cities contained 44% of the population in China until 2008^41,51,56^. Above all, urbanisation and industrialisation should be considered as important challenges leading to deforestation in Guangxi.

After 2000, the great demand for timber products, the high ecological value of eucalyptus plantation^55^ and the large-scale development of eucalyptus and logging processes also led to the conversion of woodland into grassland. Therefore, it is important to control the demand of timber products through the use of several methods, for example, reducing the use of one-off chopsticks and preventing the waste of paper^57^.

Deforestation and afforestation continued to co-exist in the two periods. During this period, approximately 30% of the afforestation changed from cropland, and 65% was converted from grassland from 2000 to 2017; at the same time, the conversion of woodland to cropland was effectively stopped after 2000. In addition, cropland—especially on slopes greater than 15 degrees—was converted into woodland after 2000 (see Fig. 7). Our study also indicates that cropland with slopes greater than 15 degrees was the primary type of converted cropland in Guangxi. This result was mainly due to the forest sustainable development policy called the “Grain for Green Program” (GFGP) that was launched in China.

Since 1999, in a bid to combat serious ecological issues, such as droughts and floods, the Chinese government launched the “GFGP” program in 25 provinces, which covers 82% of the total land area of China^45,58^. The program aimed to restore the forest cover by changing steep-sloped (many previous studies and regulations suggest slopes greater than 15 degrees) and degraded cropland and barren land into forest and grassland^45,59,60^. The GFGP is renowned as the largest ecological policy in the world and is closely related to LUCC^45,61^. Many previous studies have reported that the GFGP has worked effectively in China, as woodland has been restored^60^, ecosystem services have improved^62^, soil organic carbon levels have increased^45^, and biodiversity has been enhanced^61^. In addition, the afforestation process, including the conversion between grassland and woodland in Guangxi, can mostly be explained by the rapid growth of eucalyptus logging.

#### The ecological effects of deforestation and afforestation in Guangxi

Although the conversion from cropland to woodland has been effectively stopped, especially since 2000, the area of deforestation has decreased but is still larger than the area of afforestation. At the same time, deforestation and afforestation have co-existed in Guangxi in all periods, and this is in accordance with the results of previous studies^63^. Hansen *et al*.^63^ stated that a collocation of gains and loss of forest can be found in China, indicating that intensive forest practices are executed in this region. This result is mainly due to the large-scale development of eucalyptus plantations in China. Eucalyptus is planted not only in Guangxi but also in most of southern China, such as Guangdong, Yunnan, Hainan, Sichuan and Fujian provinces, of which approximately 1.4 million ha are in Guangxi^64^. Eucalyptus plantations provide a large amount of timber and, due to their adaptability and stable rapid growth, have caused a set of potential ecological issues, such as water deprivation, biodiversity loss, desertification, and fertilizer consumption^55,65^. In addition, our study also indicates that the conversion of steep-slope (i.e., more than 15 degrees) land is the primary conversion process in Guangxi, but the converted cropland accounts for 1.18% of the total steep-sloping cropland. As previous studies and GFGP regulations suggest, all steep-sloping cropland (especially slopes >25 degrees) should be converted to forest^47^. Thus, in terms of sustainable development in Guangxi, reducing the ecological issues of water and fertilizer related to eucalyptus plantations and implementing the GFGP are important features for the future.

As a comparison, we also used the Global Forest Change data (GFCD) (https://earthenginepartners.appspot.com/science-2013-global-forest/download_v1.5.html) to analyse the deforestation and afforestation in Guangxi; the major result found that deforestation and afforestation are collocated in Guangxi, which is in agreement with the results of a previous study^63^. However, due to the different interpolation methods, classification systems and definitions of forest (e.g., woodland in this study), there are also some different results that can be extracted from these two datasets (GFCD and CNLCD). Overall, this study analysed deforestation and afforestation based on land-use change analysis and indicated the different patterns of deforestation and afforestation. We then analysed the relationships with major policies and major forest practices, providing useful information for decision-makers.

## Conclusions

LUCC analysis can provide important information for global environment change and sustainable development studies, which can also be useful for decision-makers. In this research, using a long-time-series of (1990, 2000, and 2017) remote sensing images with high resolution (30 m and 8 m), we obtained detailed information of LUCC and highlighted the woodland conversion process in Guangxi during the periods of 1990–2000 and 2000–2017. Our findings show that the area of land use and land cover transition in Guangxi was 4708 km^2^ (2.0% of the total area), which included the six investigated land-use types. The general pattern of LUCC in this region included an expansion of built-up land and water bodies as well as a reduction in woodland, cropland, and grassland areas. The transition from cropland and forest to built-up land in addition to the transition from cropland and grassland to forest have been the dominant LUCC patterns over the past 27 years. Woodland is the dominant land-use type in Guangxi. From 1990 to 2017, deforestation and afforestation have both occurred, with deforestation being the major ecological problem during the past 27 years. After the implementation of the GFGP in 1999, the conversion from woodland to cropland was effectively curbed in this region. At the same time, the large-scale development of eucalyptus plantations has accelerated the afforestation and deforestation processes in Guangxi. Eucalyptus plantations have also led to serious ecological issues in Guangxi. Sustainable woodland management under new economic development plans should remain high on the political agenda.

This paper found the major trends of historical land-use conversions in Guangxi and indicated the related policy framework for woodland transition processes. We have provided an understanding of the national policy aspect; however, the LUCC resulted from land-use change and feedbacks on the drivers of land-use change. Therefore, a further study focused on the “cause-response” of LUCC is needed in this region.

## Materials and Methods

### Study Area

**Figure 8:**
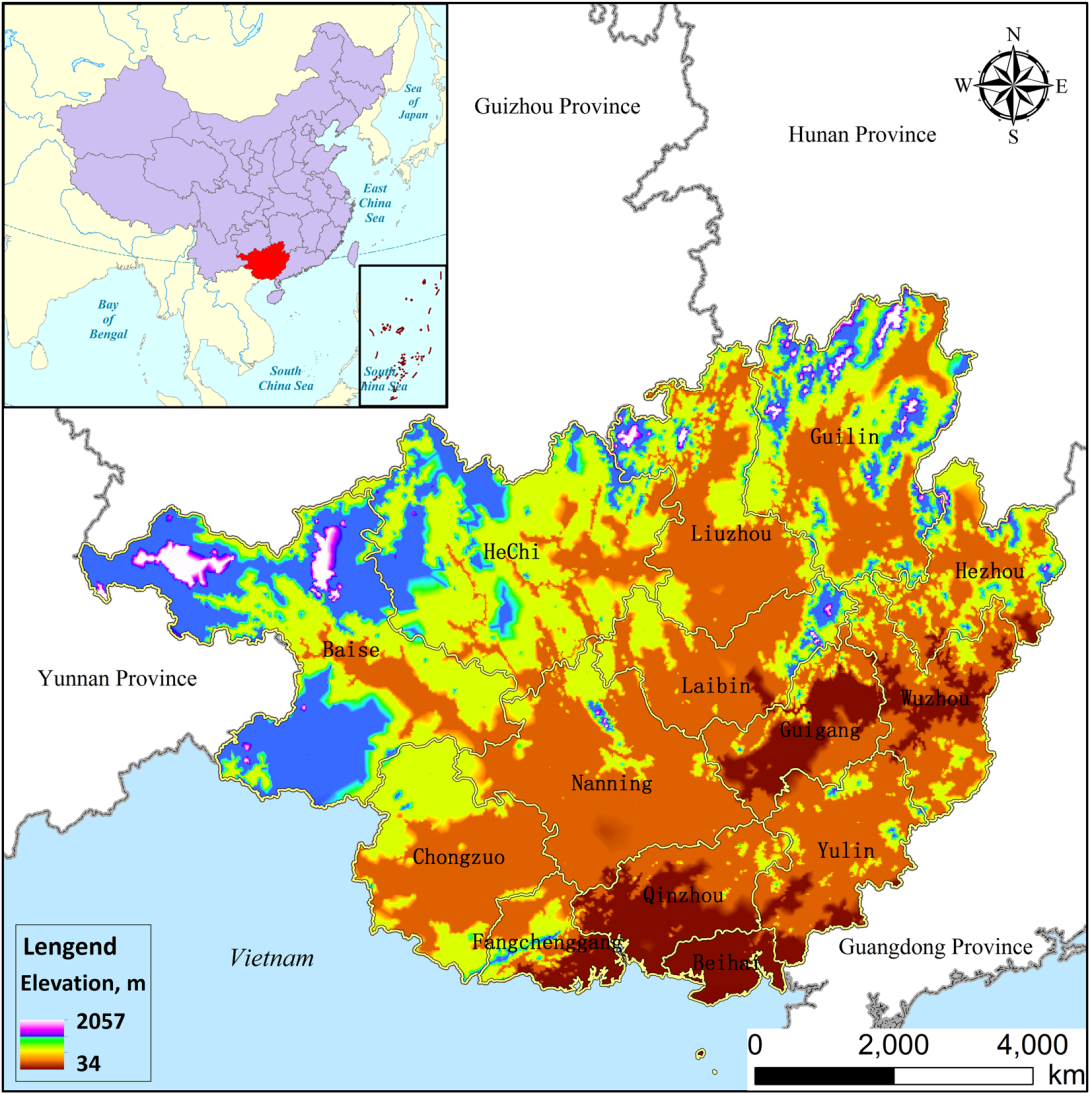
The location of Guangxi.

Guangxi, located in southern China, is a typical area that faces many ecological problems and challenges^43^. It includes fourteen municipalities (Fig. 8). Guangxi spans from 104°26′ to 112°04′ and from 20°54′ to 26°24′, covering a total area of 23.16 × 10^4^ km^2^, which is approximately 2.5% of the total area of China. The topography of the study area consists of mountains and plateaus around a flat central section, which is known as the “Guangxi Basin”^66^. Guangxi features a sub-tropical monsoon climate with an average annual temperature of 16.5–23.1 °C. The extreme highest temperature is 33.7–42.5 °C, while the extreme lowest temperature between −8.4 and 2.9 °C^67^. The climate in this region is characterised by warmth, rich heat, plentiful rainfall, and moderate sunshine, with annual wet and dry seasons^67^. Guangxi has abundant water sources because it is thickly crossed by rivers and has a 1500 km coastline bordering its southern region. This feature has led to incentives for development related to water sources, such as ocean tourism, marine chemistry, fishing, and marine resources. At the end of 2017, the permanent resident population was 48,850, which was an increase of 0.97% compared with that of 2016^68^. The regional gross domestic product (GDP) was 20,396.25 billion Yuan (CNY), with an annual increase of 7.3%^69^.

The major land-use type in Guangxi is woodland, with the proportion of woodland being over 65.5% of the total area in 2017. This is followed by cropland and grassland, which cover proportions of 21.5% and 8.8% of the total area, respectively. Most of the woodland is distributed in Baise, Hechi, and Guilin, followed by Liuzhou, Nanning, Chongzuo, and Wuzhou, with approximately 26.1% of the total woodland distributed over these seven municipalities. Beihai is the smallest woodland city, contributing only 0.7% of the total area of woodland in 2017. The total area covered by water bodies, built-up land, and unused land accounts for less than 10% of the land area in Guangxi.

### Data Collection and Processing Method

The long-time-series land-use data were obtained from Chinese National Land use/Land Cover Database (CNLCD), which was developed by the Chinese Academy of Sciences (CAS). The database spans from 1990 to 2015 and is updated at 5-year intervals. This database was produced via long-time-series remote sensing data. More than 500 TM/ETM+/OLI scenes from satellite images were used to map all of China for each period^70^. Computer-assisted visual interpretation of satellite images was used to create a digital LUCC map at a spatial scale of 1:100,000^3^. More technological details can be found in previous studies^19,21^. Then, the interpretations of the satellite images were validated against a number of field surveys. Many previous studies reported that the overall accuracy was over 95%; specifically, accuracy was 98% for grassland, forest, and built-up land and 99% for cropland^21,71^. In the present study, we created 299 random points and using Google Earth (https://www.google.com/earth/) to validate land-use data from 2017, and the overall accuracy in the first class was 89%.

These datasets have been widely used to identify the detailed information of LUCC^19,20,72^ and to identify the drivers of LUCC^21,70^, through which optimal land-use strategies can be modelled^73^.

**Figure 9:**
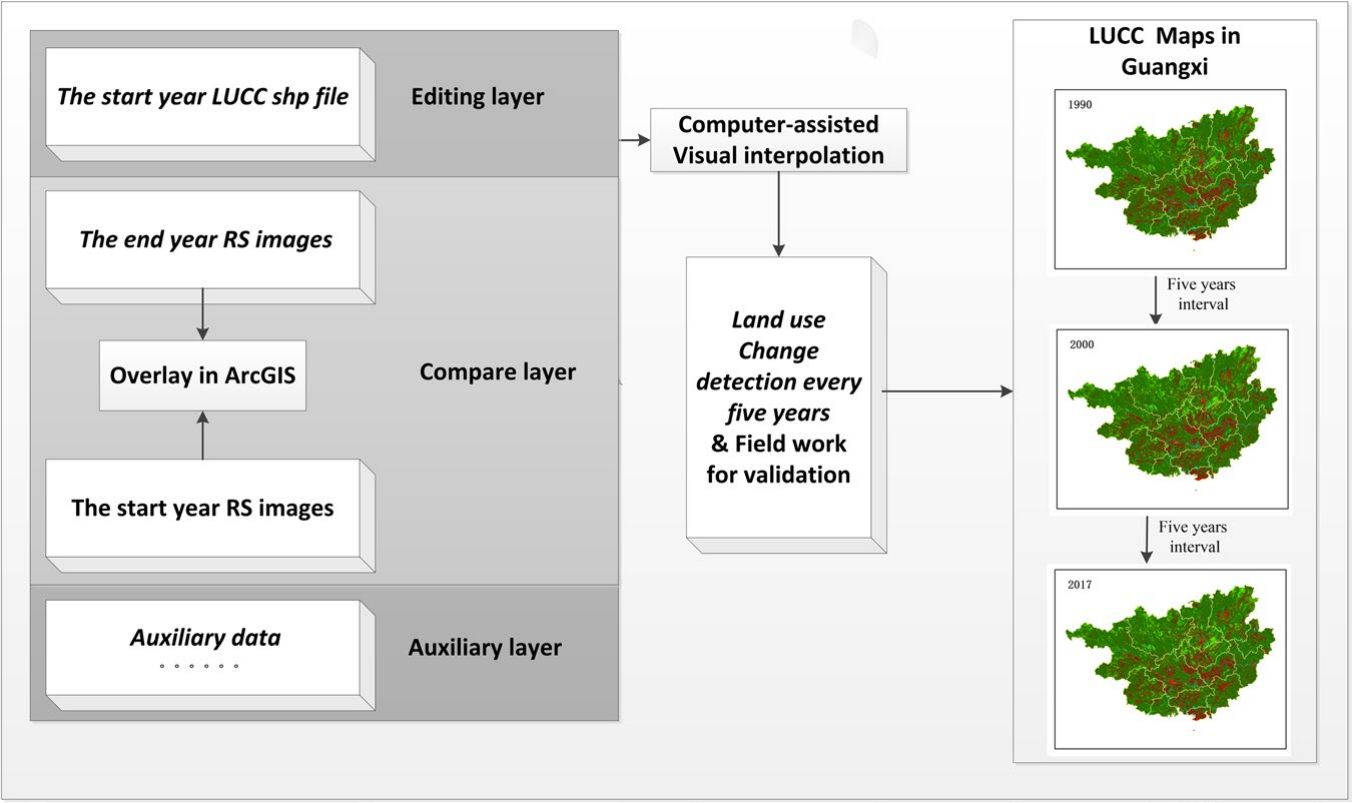
Computer-assisted visual interpretation process.

In this study, to determine the latest LUCC conversion process, our team updated the LUCC data in 2017 based on the LUCC data in 2015. The remote scenes from Landsat TM/ETM+/OLI (https://www.usgs.gov/) for 2015 and Gaofen-1 (GF-1) (http://www.cresda.com/EN/satellite/7155.shtml) for 2017 were used as the key information sources. Most of the image data were acquired during similar seasons (July–October) and were selected on the grounds of being cloud-free. Above all, the land-use data in this research covered three time periods: 1989/1990, 2000, and 2017 (see Fig. 9). The spatial resolution showed an important influence on the data accuracy^62^. However, the remote sensing images had different resolutions (30 m for 1990 and 2000, 8 m for 2017), and in an effort to create a uniform spatial scale, we interpolated based on different minimum patches. Specifically, in 1990 and 2000, 8 × 8 pixels (approximately 240 × 240 m on the ground) were employed, and in 2017, 30 × 30 pixels (240 × 240 m) were employed. For more details, refer to previous studies^74,75^. The classification used in this study was based on 25 land-use types defined by Liu *et al*.^3^, which were then grouped into six land-use types, including cropland, woodland, grassland, water body, unused land, and built-up land (Table 5).

**Table 5:** The land-use classification system.

Class	Code	Description
Cropland	1	Land for growing crops.
Woodland	2	Land for growing trees, arbor, shrubs, bamboo, and employed for forestry use.
Grassland	3	Land for herbaceous plants.
Water body	4	Natural water areas, including constructed reservoirs as well as water reservation and irrigation facilities.
Built-up land	5	Human settlements, including urban and rural settlements as well factories and transportation facilities.
Unused land	6	Land that is not put into practical use or that is difficult to use.

### Land-Use and Land Cover Change Analysis

#### Land-Use Transition Matrix

The land-use changes were calculated using a land-use transition matrix. This approach is used to describe the conversion size of land-use types in different periods^12^. The matrices of land-use transition were established for different periods in this study, including 1990–2000 and 2000–2017. The transition matrix indicates the amount of different land-use types that remain unchanged and change in the study periods. A further calculation based on the matrix table was made in an attempt to calculate the gains and losses. A gain in one land-use type is equivalent to the increase in the land-use type between the study periods, while a loss is the measured size of the decrease in that land-use type between periods^76^. For example, the gains or losses of land-use type i stand for the other land-use types having changed into *i* or land-use type *i* having been converted into other land-use types, respectively.

#### Markov chain model

Land-use change is not a unidirectional process, and a specific land-use type is theoretically changed into or from other land-use types^77^. Markov chain models are models that describe the probabilistic movements of one individual in a system from one state to the next state^78^. The land-use transition possibilities (TPs) between different states are recorded in a transition matrix that originates from the land-use transition matrix^77,79^. The transition possibilities (TPs) matrix can be expressed as follows^77^:

1 $${\rm{P}}={{\rm{P}}}_{ij}=[\begin{array}{cccc}{{\rm{P}}}_{11} & {{\rm{P}}}_{12} & \cdots  & {{\rm{P}}}_{1{\rm{n}}}\\ {{\rm{P}}}_{21} & {{\rm{P}}}_{22} & \cdots  & {{\rm{P}}}_{2{\rm{n}}}\\ \cdots  & \cdots  & \cdots  & \cdots \\ {{\rm{P}}}_{{\rm{m}}1} & {{\rm{P}}}_{{\rm{n}}2} & \cdots  & {{\rm{P}}}_{{\rm{mn}}}\end{array}]\,({\sum }_{j=1}^{{\rm{n}}}{{\rm{P}}}_{{\rm{ij}}}=1,\,0\le {{\rm{P}}}_{\mathrm{ij}}\le 1)$$

where P stands for the probability of transitioning from state i to state j, and m and n are land-use types.

The Markov chain model is a random process; the state of one land-use type at time t + 1 only depends on the current time t and is not related to any time before t. The state of the land-use type (St) at beginning time t and end time t + 1 (St + 1) can be expressed as follows:

2 $${{\rm{S}}}_{{\rm{t}}+1}={{\rm{P}}}_{{\rm{ij}}}\times {{\rm{S}}}_{{\rm{t}}}$$

#### Spatial Autocorrelation

This study used Moran’s I to analyse the global spatial autocorrelation of each land-use type in Guangxi since 1990. Spatial autocorrelation is generally used to describe and compare the spatial structure of data^80^. The basic principle of Moran’s I is as follows^80^:

3 $${\rm{I}}=\frac{{\rm{n}}}{{\rm{S}}0}\frac{{\sum }_{{\rm{i}}}^{{\rm{n}}}{\sum }_{{\rm{j}}}^{{\rm{n}}}{{\rm{W}}}_{{\rm{ij}}}({{\rm{x}}}_{{\rm{i}}}\,j\bar{{\rm{x}}})}{{\sum }_{{\rm{i}}}^{{\rm{n}}}({{\rm{x}}}_{{\rm{i}}}\,j\bar{{\rm{x}}}){({{\rm{x}}}_{{\rm{i}}}j\bar{{\rm{x}}})}^{2}}$$

where for i ≠ j, n is the total amount of the space unit; Xi is the observation value in the space unit i; $\bar{{\rm{x}}}$ is the average value of Xi; and Wij is the spatial weight matrix that represented the neighbourhood relation of area i and area j and can be measured by the distance criterion of the adjacency criterion. The adjacency is expressed as follows:

4 $${\rm{Wij}}=\{\begin{array}{c}1,\,{\rm{area}}\,{\rm{i}}\,{\rm{and}}\,{\rm{area}}\,{\rm{j}}\,{\rm{are}}\,{\rm{adjacent}}.\,\\ 0,\,{\rm{area}}\,{\rm{i}}\,{\rm{and}}\,{\rm{area}}\,{\rm{j}}\,{\rm{are}}\,{\rm{adjacent}}.\,\end{array}$$

The value of Moran’s I is between [−1, 1]. When I = 0, there is no autocorrelation between the data; when I > 0, there is a positive relation between observations; when I < 0, the relation is positive^81^. In this study, Geoda was used to calculate the global spatial autocorrelation^82^.

#### Deforestation Analysis

To highlight the features of deforestation and afforestation over time, we analysed the deforestation and afforestation rates by calculating the change rate for woodland (*k*) and mapping the spatial patterns of woodland losses and gains. The deforestation and afforestation rates were calculated using the following formula:

5 $${\rm{k}}={\rm{Sb}}-{\rm{Sa}},$$

where *Sa* and *Sb* are the areas of the land-use type at the beginning and end of a period, respectively.

## Supplementary information

The land use conversion process between 1980s and 2000 (left), between 2000 and 2017 (right)
