# Supplementary material for: Assessment of Land-Use and Land-Cover Change in Guangxi, China
Source: Sci Rep. 2019 Feb 18;9:2189. doi: 10.1038/s41598-019-38487-w (PMC6379481; doi:10.1038/s41598-019-38487-w)
Supplement: Supplementary file 1 — The land use conversion process between 1980s and 2000 (left), between 2000 and 2017 (right) [file 41598_2019_38487_MOESM1_ESM.pdf]

Assessment of Land-Use and Land-Cover Change in Guangxi, China

Yunfeng Hu[1,2]*, Batunacun[1,2,3,4]*, Lin Zhen[1,2], Dafang Zhuang[1]

[1] State Key Laboratory of Resources and Environmental Information System, Institute of Geographic Sciences and Natural Resources Research, Chinese Academy of Sciences, Beijing 100101, China
[2]University of Chinese Academy of Sciences, Beijing 100049, China
[3] Department of Geography, Humboldt–Universität zu Berlin, Unter den Linden 6, 10099 Berlin, Germany;
[4] Leibniz Centre for Agricultural Landscape Research (ZALF), Eberswalder Straße 84, 15374, Müncheberg, Germany
*Correspondence: huyf@lreis.ac.cn. batunacun@zalf.de.

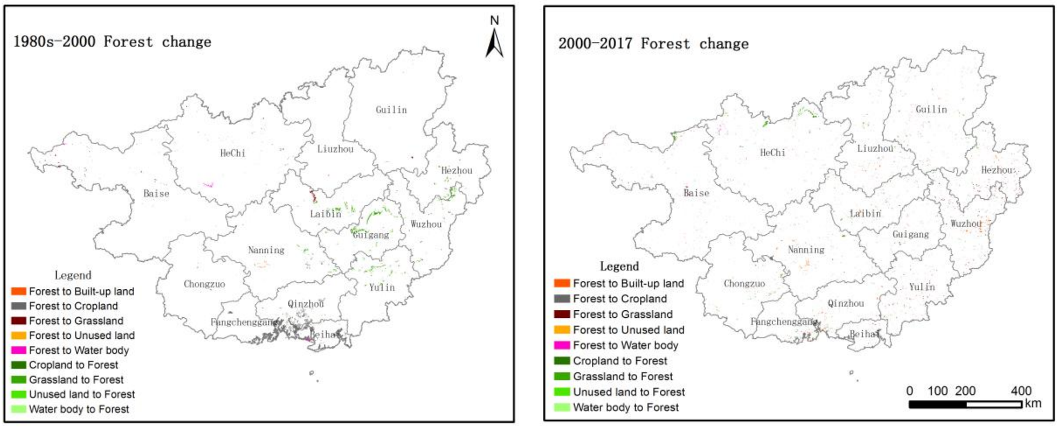

Supplementary Figure 1. The land use conversion process between 1980s and 2000 (left), between 2000 and 2017 (right)
